# Severe Fever with Thrombocytopenia Syndrome: Japan under Threat from Life-threatening Emerging Tick-borne Disease

**DOI:** 10.31662/jmaj.2019-0073

**Published:** 2020-09-30

**Authors:** Andy Crump, Tetsuya Tanimoto

**Affiliations:** 1 Kitasato University, Tokyo, Japan; 2 Medical Governance Research Institute, Tokyo, Japan

**Keywords:** SFTS, tick-borne virus, emerging disease

## Abstract

Japan, like many other parts of the world, is under threat from newly emerging, potentially fatal diseases. Severe fever with thrombocytopenia syndrome (SFTS), first clinically identified in 2009, is an emerging tick-borne hemorrhagic viral disease, currently limited in distribution to East Asia. Relatively little is known about the disease with an initial Case Fatality Rate ranging from 5% to 40%. It primarily affects the elderly living in rural areas, which is particularly troublesome given Japan’s rapidly aging population. Control efforts are severely hampered by lack of specific knowledge of the disease and its means of transmission, coupled with the absence of both a vaccine and an effective treatment regime, although some antiviral drugs and blood transfusions are successful in treating the disease. Despite both the causative virus and vector ticks being commonly found throughout Japan, the disease shows a very specific, limited geographical distribution for as yet unknown reasons.

## Introduction

Having little or no knowledge of the novel diseases or how to prevent, diagnose, and treat them, new, potentially fatal diseases that suddenly appear pose a huge dilemma for doctors and health services. The rapid worldwide spread of new tick-borne diseases (TBDs) has recently become of profound public health concern. In the last decade, Japan has experienced the advent of the potentially fatal severe fever with thrombocytopenia syndrome (SFTS), a TBD about which comparatively little is known and for which no specific drugs exist and no therapeutic protocol has yet been determined.

Ticks are small blood-feeding arachnids that can transmit a variety of infectious agents, including viruses, bacteria, and protozoan parasites. The pathogens they transmit cause the vast majority of vector-borne human diseases in temperate Asia, Europe, and North America ^(1)^. Since the turn of the century, the incidence of tick-borne illnesses has been steadily increasing, with the geographic areas in which they exist both expanding and shifting in line with changing tick habitats and tick distribution continuing to expand northward in latitude and upward in elevation in both Europe ^(2)^ and North America ^(3)^. Consequently, health workers increasingly face the impossible task of distinguishing the diverse clinical symptoms of these emerging diseases, which they often encounter for the first time.

For the past few years, Japan has been confronted with a particularly complex TBD problem. In 2016, a 50-year-old Japanese woman died 10 days after being bitten by a stray cat. The Ministry of Health, Labour and Welfare confirmed that the cause of death was SFTS, an emerging viral disease, first identified in 2009, which was believed to be transmitted by ticks^(4)^. However, with no tick bite detected on the woman, doctors assumed the disease had somehow been contracted from the cat, despite the fact that no animal-to-human transmission of the virus had previously been reported. As it continues to spread, SFTS is attracting increasing global attention as relatively little is known about the disease and no standardized treatment currently exists. In 2015, the World Health Organization compiled a list of priority pathogens with the potential to generate an international public health emergency, for which no, or insufficient, preventive or curative solutions existed. An initial list of seven diseases was decided, with three others, namely, SFTS, chikungunya, and zika, designated to be both serious and necessitating urgent action as soon as possible ^(5)^. SFTS was subsequently moved onto the list after the annual review in 2016 ^(6)^.

The scope and magnitude of tick-borne infections have been increasing worldwide over the past 30 years. Epidemiologically significant diseases transmitted by ticks have correspondingly increased. In some regions, such as Europe, TBDs are now the most widespread and medically important of all vector-borne infectious diseases ^(7)^. In Asia, SFTS is an emerging infectious hemorrhagic disease that exhibits high Case Fatality Rates (CFR) in humans (5%-40%) and is especially severe in people over 50, thus posing a particular threat to Japan’s rapidly aging population.

## Causative Agent

SFTS was first discovered in Northeast China in 2007, its main clinical symptoms were identified in 2009, and the causative virus was isolated in 2010 ^(8), (9)^. The disease was subsequently detected in Japan in 2012 and South Korea ^(10), (11)^ and is now known to be fairly widespread in East Asia ^(12)^. Consequently, knowledge of the virus and disease is still relatively scant, hindering attempts to implement effective control measures. Reflecting its relatively recent emergence, the virus that causes SFTS has already been reclassified several times. According to the International Committee on Taxonomy of Viruses, the virus species is *Dabie bandavirus *in the Phenuiviridae family. However, the initial SFTSV acronym is still commonly used. Research findings suggest that this virus may actually have originated in China between 50 and 150 years ago but that, based on genetic analyses, it is likely that it originated in Japan and Korea soon after its emergence ^(13)^. Phylogenetic analysis shows that Japanese viral strains form a cluster distinct from other strains, indicating that the virus has evolved independently in Japan.

SFTSV appears to be able to maintain itself extremely well in nature. It can infect many mammalian hosts, including cats, mice, dogs, goats, cattle, chicken, wild birds, weasels, and yaks, with such animals possibly acting as reservoir hosts ^(14), (15), (16), (17)^. In Japan, a high prevalence of anti-SFTSV antibody seropositivity has also been recorded in deer, wild boar, and raccoons, although this does not mean that they are involved in the circulation of the virus ^(18)^. Researchers have also suggested that migratory birds may play a role in spreading the virus ^(19)^. Humans appear to be accidental dead-end hosts, playing no role in the SFTSV life cycle. The virus life cycle and natural mechanisms of sustained transmission remain poorly understood, but transmission to humans via tick bites is deemed the most probable route ^(20), (21), (22), (23)^. Several SFTSV vector tick species have already been identified, including *Haemaphysalis longicornis*,* Amblyomma testudinarium*, *Ixodes nipponensis*, and *Rhipicephalus microplus* ^(24), (25)^. In addition, in Japan, *Haemaphysalis flava*, *Haemaphysalis megaspinosa*, and *Haemaphysalis kitaokai *are suspected to be potential SFTSV vectors ^(26)^. Nevertheless, in a Japanese study, 21 (44%) of the 48 patients had no trace of any tick bite. It is now believed that SFTSV can be transmitted from person to person through contact with blood or mucus from an infected individual ^(27)^. It has also been found in semen, meaning that the SFTSV could be sexually transmitted ^(28)^. The virus has been detected in companion animals, livestock, and other wild mammals, meaning elderly rural dwellers with livestock or domestic pets may be particularly at risk. 

## Disease

The precise clinical characteristics and primary risk factors for SFTS have not yet been fully clarified in Japan. Patients tend to be elderly, outdoor workers in rural areas or retired, unemployed people undertaking outdoor pursuits, such as hiking or camping, in areas and environments where ticks thrive. Following infection, the incubation period is 5-14 days with an average of 9 days ^(12)^. The potentially fatal viral hemorrhagic disease is initially characterized by nonspecific symptoms. Early-stage indications are fever, gastrointestinal symptoms (anorexia, nausea, vomiting, etc.), headache, and myalgia. Disease progression is marked by leukopenia, thrombocytopenia, liver dysfunction, neurological symptoms (impaired consciousness), bleeding (gingival seepage, bloody diarrhea, and hematuria), and hemophagocytic syndrome, eventually leading to disseminated intravascular coagulation and multiple organ failure ^(8), (9), (10), (11), (12)^. Virological diagnosis follows SFTSV detection in a patient’s blood or other bodily fluids (e.g., urine, mucus, semen). Diagnostic laboratory testing looks for lymphopenia and thrombocytopenia in total blood cell count (TBC) and elevated levels of serum aminotransferases (AST and ALT) and lactate dehydrogenase in blood serum. Confirmatory diagnosis uses RT-PCR detection of SFTSV genomic material or indirect fluorescent antibody testing using SFTSV-infected cells and IgG-ELISA using SFTSV antigen. In survivors, TBC improves about 1 week after onset, returning to normal within 2 weeks ^(26)^.

## Status in Japan

**Figure 1.**
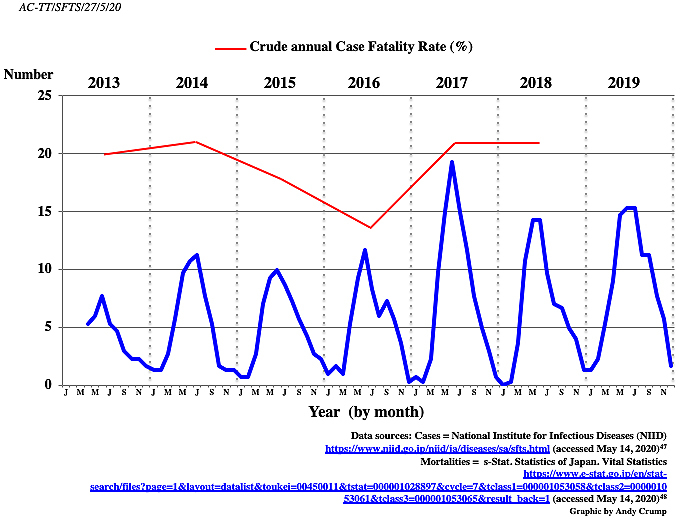
Japanese SFTS cases reported by month (2013-2020) (three-point moving average).

In Japan, the list of notifiable diseases (infectious diseases Categories I and IV) includes 12 tick- or mite-borne diseases: Japanese spotted fever, Crimean-Congo hemorrhagic fever, Lyme disease, Rocky Mountain spotted fever, relapsing fever, tularemia, scrub typhus, Q-fever, Omsk hemorrhagic fever, Kyasanur forest disease, tick-borne encephalitis, and SFTS. Since 2013, SFTS has been designated as a Category IV disease under Japan’s Infectious Diseases Control Law, mandating physicians to notify all laboratory-confirmed cases to the government ^(29)^. Following discovery, Japan’s National Institute for Infectious Diseases figures show that there were 498 Japanese SFTS cases reported up until 2020. In the first year of reporting (2013), 40 cases were identified, although there is solid evidence that at least 8 cases had occurred prior to this. Of the 40 cases identified in 2013, it has long been thought that 35% proved fatal, although our analysis of more reliable data indicates that the figure may well have been only 20% (Table 1). Although tick-borne, it has been confirmed that humans can become infected with SFTSV from companion animals, such as cats and dogs. In Japan, a case of suspected human-to-human SFTSV transmission among family members has been recorded, while health workers have been infected by patients in both China and Korea ^(30)^. In Japan, disease cases have so far followed a seasonal pattern (Figure 1), with the majority appearing in the summer months. When Japan is hot and humid, ticks are most active and individuals work intensively or pursue outdoor activities in rural areas. However, currently, there is no explanation as to why the disease seems to affect only a certain part of the country.

**Table 1.** Japanese SFTS Cases and Fatalities (March 2013-December 2019).

Cases	2013	2014	2015	2016	2017	2018	2019	Total
Total	40	61	60	60	90	77	102	490
Dead	8	13	11	8	19	16	n/a	75*
CFR(%)	20	21	18	13	21	21		19**

Data of eight retrospective cases identified prior to official records beginning in March 2013 not included. CFR = Case Fatality Rate * Number for 2013-2018 inclusive ** Number based on data from 2013-2018 inclusive Data sources: Cases = National Institute for Infectious Diseases (NIID) https://www.niid.go.jp/niid/ja/diseases/sa/sfts.html (accessed May 14, 2020) ^(47)^ Mortalities = e-Stat. Statistics of Japan. Vital Statistics https://www.e-stat.go.jp/en/stat-search/files?page=1&layout=datalist&toukei=00450011&tstat=000001028897&cycle=7&tclass1=000001053058&tclass2=000001053061&tclass3=000001053065&result_back=1 (accessed May 14, 2020) ^(48)^ Graphic by Andy Crump

## Distribution

The risk of contracting a tick-borne infection depends on a combination of the overall number of ticks in an area (tick density), the proportion of ticks in the location that carry a disease-causing agent (tick infection rate), and human behavior (exposure). People engaged in recreational or occupational outdoor activities in a high-risk area are particularly vulnerable to infection. Like all vector-borne diseases, tick distribution is determined by a complex set of interacting factors. Changes in agricultural practices as a result of changes in temperature and rainfall can affect transmission. Global travel and trade, unplanned urbanization and environmental disturbances, such as climate change, can also influence pathogen transmission, possibly extending the transmission season or causing diseases to emerge in countries where they were previously absent. Climate is known to play a major role in governing the geographic localization of disease vectors, including ticks. Recently, the frequency and geographic areas at risk of tick-borne infectious diseases have been changing and expanding, possibly due to climate change ^(1), (7), (31), (32)^.

**Figure 2.**
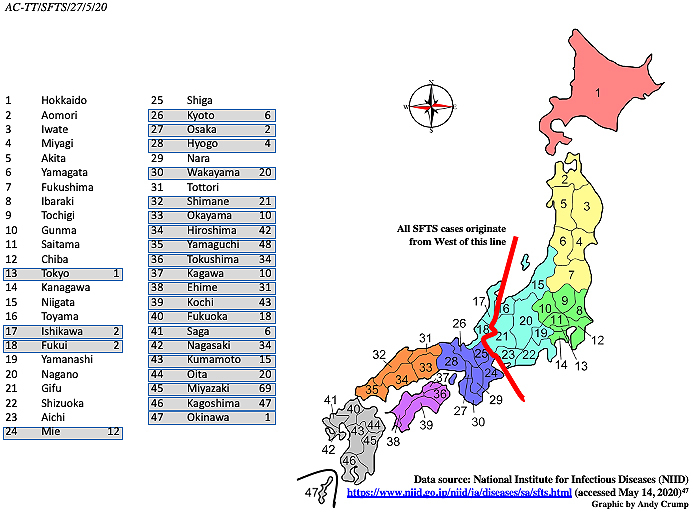
Geographic distribution of SFTS cases in Japan by prefecture (2013-2019 inclusive).

In East Asia, specific mountainous regions of China and western Japan exhibit higher frequencies of SFTS notifications. Altitudes of 80-400 m together with several climatological variables, including average temperature, humidity, and precipitation, as well as livestock density, are associated with an increased risk of tick-borne infections ^(33), (34)^. Meteorological factors that may influence the growth and development of tick vectors and the daily rhythms and behavior of at-risk people, such as temperature, precipitation, and sunlight duration, affect the incidence of SFTS. Epidemiological data imply that, in Japan, confirmed cases are most commonly observed among elderly retired or unemployed farmers (average age, 78 years) who live in rural regions or near forested areas or among people who go hiking or any other activity that takes them into environments where they may be exposed to ticks ^(35)^. Yet, in reality, comparatively little is yet known about why the disease in Japan exhibits its current distinctly limited distribution (Figure 2). Japan is an elongated, thin country, ranging from subarctic in the north to subtropical in the south, generally with four distinct seasons. Conditions are also significantly different between the Pacific side and the Sea of Japan side. Northern Japan has a humid continental climate with long, cold winters and very warm to cool summers. Precipitation is not heavy, but winter sees heavy snow on the Sea of Japan side and in mountainous areas. Eastern Japan has hot and humid summers and long cold winters with heavy snow on the Sea of Japan side and in mountainous areas. Western Japan has very hot and humid summers and moderately cold winters. The southern regions of Okinawa and Amami have a typical subtropical oceanic climate. Surprisingly, with respect to SFTS, there is an apparent presumably climatic component that appears to influence the geographic location of the disease, but not the virus. SFTSV, as well as tick species capable of transmitting SFTSV, is found throughout Japan. Even though ticks from parts of the country with no SFTS cases contain detectable amounts of the SFTSV genome, disease cases so far have been significantly restricted to localities in the southwestern parts of the Japanese islands (Figure 2). So far, the disease has been reported from 24 of the nation’s 47 prefectures, with Miyazaki prefecture being the worst affected. Studies there have concluded that the risk of SFTS is highest in areas at mid-level altitudes where land is not used intensively for farming and, especially, in flatlands near hilly locations along riversides ^(36)^.

## Impact

In China, the incidence rate of SFTS was significantly higher in elderly people and fatal cases mainly occurred in people of advanced age ^(16)^. As regards the impact on the Japanese population so far, the disease also seems to be primarily occurring in elderly individuals, with an average age of 75, and is found equally in males and females (Table 2). The average age of those who have died from the disease is 81.5 years. Unfortunately, to date, the annual CFR has been sustained at a level of around 20% (Figure 1), despite the improving knowledge of the disease, earlier detection, and better health service interventions. The initial crude annual CFR in 2013 (calculated by the number of deaths (8) divided by the number of cases (40)) of 20% stood at 20.8% (16/77) in 2018. For the entire period (2013-2018), the CFR was 19% (Table 1).

**Table 2.** Japanese SFTS Cases by Sex, Age, and Fatalities (2013-2019 Inclusive).

		Alive	Dead	Total
Total		428	70	498
Sex	Male	207	37	244
	Female	221	33	254
Age (years)	0-29	5	0	5
	30-39	8	0	8
	40-49	12	0	12
	50-59	28	3	31
	60-69	106	11	117
	70-79	135	18	153
	80-89	116	33	149
	90+	18	5	23
Average age		74	81.5	75

Data source: National Institute for Infectious Diseases (NIID) https://www.niid.go.jp/niid/ja/diseases/sa/sfts.html (accessed May 14, 2020) ^(47)^ Graphic by Andy Crump

## Treatment Options

Currently, a vaccine for disease prevention is not available and no standardized treatment protocol has been established yet for SFTS. To date, no specific medicaments have been identified, although some existing antiviral agents have been used effectively. Supportive treatment, such as blood transfusion, renal replacement therapy, and empirical antibiotics, has so far been the fundamental intervention ^(37), (38)^. In China, intravenous ribavirin has been a guideline treatment for SFTS ^(39)^. Ribavirin inhibits the replication of SFTSV *in vitro*, although its efficacy in a clinical setting is relatively limited ^(40)^. Research indicates that favipiravir, developed by the Japanese Toyama Chemical company, may be a better option. Favipiravir is a potent broad-spectrum antiviral drug that inhibits the replication of multiple families of RNA viruses *in vitro* and *in vivo*. It is a therapeutic antiviral drug approved in Japan for use against novel or reemerging influenza. Favipiravir displays a wide range of anti-RNA virus activities *in vitro* and has proved effective against several lethal RNA viruses in animal models. It has already been used for the treatment of human infection with life-threatening viruses such as those that cause Ebola hemorrhagic fever, Lassa fever, and rabies ^(41)^. Several clinical trials investigating the efficacy of favipiravir in SFTS patients are underway in Japan, but the results are not yet published. However, favipiravir given to two Japanese SFTS patients brought about complete remission ^(42)^. The drug has also displayed activity against several newly discovered viruses, such as the Heartland virus (HRTV) ^(43)^ and Severe Acute Respiratory Syndrome Coronavirus 2 ^(44), (45), (46)^. HRTV is another emerging tick-borne virus, similar to SFTSV, with HRTV-infected patients displaying similar symptoms to SFTS patients.

## Concluding Remarks

Clearly, cohesive, detailed epidemiological and clinical research is urgently needed to build up an evidence base on SFTS to better understand the clinical and pathological aspects of the disease, how it is transmitted, how it can be effectively treated, and how infection can best be prevented. Development of rapid diagnostic kits would also be extremely useful, as would development of a vaccine. However, those goals and products remain a long way off. Currently, tackling SFTS has involved responding to those infected and focusing on various comprehensive prevention and control measures, including advocacy and communication to minimize the routes of transmission to protect vulnerable individuals. The primary advice is to avoid tick bites by covering skin and using insect repellent in situations where ticks may be encountered. If the disease persists and spreads, it may become necessary to check donated blood supplies for the presence of the virus, although giving blood is not a common practice in Japan, with the number of donors steadily declining. The CFR of SFTS in Japan has remained unacceptably high, and it is becomingly increasingly obvious that specific treatment with effective antiviral agents, either novel or existing, is urgently required to reduce or overcome SFTS morbidity and mortality. At present, prevention remains the most realistic option for high-risk individuals, such as Japan’s increasingly elderly inhabitants in comparatively resource-poor rural areas, where health services are far from ideal compared to what is available to the majority of the nation’s citizens who live in densely populated urban centers.

## Article Information

### Conflicts of Interest

Tetsuya Tanimoto received personal fees from the Medical Network Systems (MNES) Inc., outside the submitted work.

### Acknowledgement

We would like to thank the Medical Governance Research Institute (MEGRI) for their assistance.

### Author Contributions

AC reviewed the literature and drafted the manuscript. All authors contributed to the concept of the manuscript, interpretation of the literature, and critical review of the manuscript and approved the final version.

### Approval by Institutional Review Board (IRB)

Not applicable.
